# Stachytine Hydrochloride Improves Cardiac Function in Mice with ISO-Induced Heart Failure by Inhibiting the α-1,6-Fucosylation on N-Glycosylation of β1AR

**DOI:** 10.3389/fphar.2021.834192

**Published:** 2022-02-08

**Authors:** Panwei Hu, Shuting Guo, Songru Yang, Sining Wang, Sai Wang, Xiaoli Shan, Pei Zhao, Wei Guo, Ming Xu, Chen Zhang, Rong Lu, Huihua Chen

**Affiliations:** ^1^ School of Basic Medical Science, Shanghai University of Traditional Chinese Medicine, Shanghai, China; ^2^ Department of Comprehensive Internal Medicine, Tongde Hospital of Zhejiang Province, Hangzhou, China; ^3^ Public Laboratory Platform, School of Basic Medical Science, Shanghai University of Traditional Chinese Medicine, Shanghai, China; ^4^ Department of Pathology, Shanghai University of Traditional Chinese Medicine, Shanghai, China; ^5^ Department of Physiology, Shanghai University of Traditional Chinese Medicine, Shanghai, China; ^6^ Teaching and Research Department of Basic Theory of Traditional Chinese Medicine, Shanghai University of Traditional Chinese Medicine, Shanghai, China

**Keywords:** heart failure, stachydrine hydrochloride, β1 adrenergic receptors, N-glycosylation, α-1,6-fucosylation

## Abstract

**Background:** Cardiovascular diseases have become a major public health problem that seriously threatens human health. The cumulative effects of various cardiovascular events will eventually develop into chronic heart insufficiency and even heart failure, and the β1 adrenergic receptor signal pathway plays an important role in this process. Stachytine hydrochloride is the main active ingredient of Yimucao, which is a traditional Chinese medicine used to treat gynecological diseases. Modern studies have found that stachytine hydrochloride has a good cardioprotective effect, but it is still unclear whether stachytine hydrochloride has an effect on the β1 adrenergic receptor signal pathway. The purpose of this study is to explore the effect of stachytine hydrochloride on the β1 adrenergic receptor signal pathway.

**Method:** In this study, a continuous infusion of isoproterenol (40 mg/kg/day) was administered to mice and ventricular myocytes explored the potential mechanism of stachytine hydrochloride (12 mg/kg/day) on the β1 adrenergic receptor signal pathway in the heart. Evaluate changes in cardiac morphology and function by echocardiography, cardiac hemodynamics, and histological methods, and detect molecular changes by Western blot and immunofluorescence. Treat primary cultured adult mouse or neonatal rat ventricular myocytes with or without isoproterenol (0.1 μMol), PNGase F (10^–2^ units/ml), and stachytine hydrochloride (10 μMol) at different time points. Detect α-1,6-fucosylation on N-glycosylation, calcium transient, contraction, and relaxation function and related signals.

**Results:** Stachytine hydrochloride reduces cardiac remodeling and modulates hemodynamic parameters during chronic β1 adrenergic receptor activation *in vivo*. The N-glycosylation of β1 adrenergic receptors decreased after continuous isoproterenol stimulation, while stachytine hydrochloride can increase the N-glycosylation of β1AR in the heart of mice with isoproterenol-induced heart failure. Decreased N-glycosylation of β1 adrenergic receptors will downregulate the cAMP/PKA signal pathway and inhibit myocardial excitation and contraction coupling. Stachytine hydrochloride significantly reduced isoproterenol-induced cardiac N-linked glycoproteins with α-1,6-fucosylation.

**Conclusion:** Our results show that stachytine hydrochloride inhibits the synthesis of α-1,6-fucosylation on the N-terminal sugar chain by reducing α-1,6-fucosyltransferase (FUT8) and α-1,3-mannosyl-glycoprotein 4-β-N-acetylglucosaminyltransferase A (MGAT4a), upregulating the N-glycosylation level on β1 adrenergic receptors, and maintaining cAMP/PKA signal pathway activation.

## Introduction

G protein-coupled receptors (GPCRs), also known as seven-pass transmembrane receptors, are the largest family of receptors on the cell membrane (Bjarnadóttir et al., 2006). They can be activated by many ligands and have become important targets for the diagnosis and treatment of various diseases. GPCRs can undergo many kinds of post-translational modifications, such as glycosylation, phosphorylation, and ester acylation. Among them, glycosylation is a very common post-translational modification of membrane proteins in living cells (Chuh et al., 2016), and N-glycosylation is the most common glycosylation modification. N-glycosylation modification may be very important for the expression and folding of membrane proteins on the cell surface (Dennis et al., 2009) and affect the expression level of many kinds of GPCRs on the cell membrane surface (He et al., 2002; Dennis et al., 2009; Soto and Trejo, 2010).

*Leonurus japonicus* Houtt. (Yimucao) is a traditional Chinese medicine. It is often used to treat gynecological diseases because it promotes blood circulation to remove blood stasis and induces diuresis to alleviate edema (Miao et al., 2019). Modern experiments and clinical studies have shown that Yimucao can relieve myocardial ischemia, increase coronary blood flow, and improve heart function. Because it can improve hemodynamics and hemorheology, it has a protective effect on protecting the cardiovascular system (Liu et al., 2012). Our research group conducted a series of studies on the effect of stachytine hydrochloride (Sta), the main active component of Yimucao, and found that Sta can inhibit norepinephrine (Zhang et al., 2014), phenylephrine (Zheng et al., 2020), and TAC-induced myocardial hypertrophy; reduce calcium leakage; maintain calcium homeostasis; inhibit myocardial fibrosis (Liu et al., 2019); and improve cardiac function (Chen et al., 2020). However, the molecular target of Sta is still unclear.

Under physiological conditions, β1AR can bind to and activate Gs protein after being activated. Then, the synthesis of cyclic adenosine monophosphate (cAMP) increases, which activates protein kinase A (PKA) and downstream signaling molecules to enhance myocardial contractility. During the development of heart failure, the continuous increase of catecholamines in the blood promotes the abnormality of the β-adrenergic receptor system. The content of β1 adrenergic receptors in the heart was significantly downregulated, the density of β1AR on the cell membrane was downregulated by 50%, while β2AR did not show this change (Bristow et al., 1982; Bristow et al., 1986; Bristow et al., 1993). Relevant studies have shown that N-glycosylation on β1AR may regulate receptor dimerization on the cell membrane surface by reducing receptor expression (He et al., 2002). Whether Sta regulates the N-glycosylation of myocardial β1AR and maintains the stable number of β1AR, thereby improving the contractile function of the heart, is the focus of this article.

## Materials and Methods

### Chemicals and Reagents

Stachydrine hydrochloride (purity > 98%) was purchased from the National Institutes for Food and Drug Control (Beijing, China). All the other drugs were from Sigma-Aldrich (St Louis, MO, United States), except for those mentioned.

### Animal Manipulation

All procedures in C57BL/6J mice and neonatal Wistar rats were approved by the Animal Care and Use Committee of Shanghai University of Traditional Chinese Medicine (SCXK2016-0011). All animal care and experimental protocols were in compliance with the *Guide for the Care and Use of Laboratory Animals* (NIH, 8th Edition, 2011). Adult male C57BL/6J mice (age, 7 weeks) were purchased from the Shanghai Laboratory Animal Center (Shanghai, China). Animals were housed in an individually vented cage system under a controlled 12-h-light/dark cycle with free access to food and water. Isoprenaline (40 mg/kg/day, ISO group) or vehicle (saline, Sham group) were infused subcutaneously to mice with mini-osmotic pumps (Alzet model 1002, Durect, Cupertino, CA, United States) for 14 days. Sta was orally administered at a constant dose of 12 mg/kg/day to the ISO-infused (ISO + Sta) group and saline-infused (Sham + Sta) group. After experiment completion, mice were euthanized by cervical dislocation. Their hearts were immediately snap-frozen in liquid nitrogen as soon as they were removed during the heart operation and stored at −80°C until use.

### Adult Mouse Ventricular Myocytes Culture and Treatment

After mice were anesthetized by intraperitoneal injection with 1% pentobarbital sodium, the Langendorff constant flow perfusion system was used to intubate aorta in mice, and then collagenase type II (#17101015, Thermo Fisher Scientific, United States) was used to perfuse heart. The myocardial tissue was cut into small pieces; thereafter, myocardial cells were obtained after repeated gentle blowing and beating. Isolated cardiomyocytes were inoculated in a 35-mm glass bottom dish wrapped with laminin (#23017015, Thermo Fisher Scientific, United States) and attached to the wall in a 5% CO_2_ incubator at 37°C for 2 h. The blank control was given with serum-free M199 medium (GIBCO, United States), while PNGase F (10^−2^ units/ml) purchased from NEB were administered to the experiment group for 2 h. After treatment, under the stimulation conditions of bright field and 1 Hz field, the motion of myocardial sarcomere was tracked by using Si-H optical sarcomere spacing system (WPI, United States) according to the change of gray value of myocardial sarcomere. The systolic and diastolic functions of single myocardial cells were calculated by Micro-Manger 1.4 software (University of California San Francisco).

### Neonatal Rat Ventricular Myocytes Culture and Treatment

Primary mouse cardiomyocytes were isolated from Wistar rats within 24 h of birth by serial enzymatic digestion. When the neonatal rats were decapitated, their hearts were quickly excised and placed in phosphate buffer saline. After cleaning and removing the atrial tissue, the ventricles were chopped with scissors and digested using a solution containing collagenase II and 0.25% trypsin (#25200072, Thermo Fisher Scientific, United States). Cells were plated on six-well plates or confocal dishes and maintained in DMEM/F12 medium (GIBCO, United States), containing 10% fetal bovine serum for 48 h at 37°C in a 5% CO_2_ incubator as described previously.

For *in vitro* experiments, in order to determine the most effective time of glycosylation, neonatal rat cardiomyocytes were exposed to isoprenaline at several time points (0, 15, 30, 60, 360, and 720 min). When the optical time of administration was confirmed, cardiomyocytes were dosed to the following groups: control group (Con), Con + Sta (10 μMol) group, ISO (0.1 μMol) group, and ISO (0.1 μMol) + Sta (10 μMol) group. In all *in vitro* assays, the cells were incubated with DMEM/F12 without fetal bovine serum for synchronization.

### Transcriptome Library Construction and Sequencing

Total mRNAs in Con and ISO groups were extracted from mouse ventricular tissue using TRIzol (Life Technologies, Carlsbad, CA, United States). The mRNA-seq libraries were prepared using the Hieff NGS^™^MaxUp Dual-mode mRNA Library Prep Kit for Illumina^®^ (YEASEN, 12301ES96, Shanghai, China); then, the Illumina sequencing platform HiSeq X Ten (Illumina, Shanghai OE Biotech Co., Ltd.) was used to sequence these libraries. HISAT2 software was selected to map the filtered sequenced reads to the reference genome, TPM (Transcripts per kilobase of exon per million mapped reads) were calculated as follows. The differential genes (DEGs) were next determined with DESeq2 R package following the standard |log FC | > 1 and adjusted *p*-value < 0.05. R package clusterProfiler were used to perform GO function enrichment analysis and KEGG pathway enrichment analysis. The data presented in the study was deposited in the NCBI SRA BioProject repository, and the accession number was PRJNA793393.

### Western Blot Assay and Antibodies

Membrane protein and cytoplasmic protein in myocardial tissue were extracted using a Mem-PER Plus Membrane Protein Extraction kit (#89842, Thermo Fisher Scientific, United States). Cardiomyocytes in a six-well plate were rinsed with phosphate-buffered saline (PBS) before being scraped and lysed in RIPA assay (#P0013B, Beyotime, Beijing, China) buffer supplemented with protease inhibitor cocktail and phosphatase inhibitor cocktail II (Roche, Mannheim, Germany). After centrifugation at 14,000 g for 20 min, 40 μg of the supernatant protein was obtained and subsequently divided into two same portions. Lysis was added into the first portion (without PNGaseF) to make the same volume with the second portion. While on the second portion, 20 µg of protein is denatured with 1x Glycoprotein Denaturing Buffer at 100°C for 10 min according to their respective processing protocols. After the addition of 10× NP-40 and GlycoBuffer two into the second group, 1 μl of PNGase F (20 μg/unit) was added and the reaction mix is incubated for 2 h at 37°C. Finally, all samples were prepared for electrophoresis by the addition of 5× loading buffer and boiled for 10 min. Then, protein lysates were separated using 10% polyacrylamide gel and transferred to polyvinylidene fluoride membranes (EMD Millipore, Billerica, MA, United States) on Mini Trans-Blot Cell (Bio-Rad Laboratories, Hercules, CA, United States) blocked with BSA (Weiao Biotechnology Co, Shanghai, China). Membranes were blocked in 5% BSA (Weiao Biotechnology Co, Shanghai, China) for 1 h at room temperature and successively incubated with primary antibodies overnight at 4°C. GAPDH was employed as the internal reference. The following primary antibodies were used in Western blot assay: Anti-beta 1 Adrenergic Receptor (β1AR) (1:1,000, ab3442, Abcam, Cambridge, CB, United Kingdom) and Anti-GAPDH (1:3,000, #97166, Cell Signaling Technology, Beverly, MA, United States). Membranes were incubated with appropriate secondary antibody for 1 h at room temperature. Secondary antibody was selected as follows: HRP Goat Anti-Rabbit IgG (1:3,000, AS014, Abclonal, Wuhan, China) or HRP Goat Anti-Mouse IgG (1:5,000, AS014, Abclonal, Wuhan, China). Signal was detected using the ECL system (Image Quant LAS 4000, Amersham Biosciences, GE Healthcare, Diegem, Belgium) according to the manufacturer’s instructions.

### Lens Culinaris Agglutinin Lectin Blot Assay and Antibodies

Protein sample preparation and SDS-PAGE procedures were performed as Western blot. The following antibodies were used in lectin blot: LCA (Biotinylated-LCA) (1:1,000, B-1045-5, VectorLab, Burlingame, VT, United States) and Streptavidin-HRP (1:3000, #3999, Cell Signaling Technology, Beverly, MA, United States).

### Immunofluorescence

The cardiac tissues in different groups were prepared into frozen sections for immunofluorescence analysis. For cellular immunofluorescence, cells were washed three times with PBS. When the samples were prepared and fixed with 4% PFA for 10 min, 5% BSA was used to block for 1 h before the addition of primary antibodies. The primary antibodies were as follows: LCA (Fluorescein-LCA) (1:1,000, FL-1041-5, VectorLab, Burlingame, VT, United States) and β1AR (1:100, ab3442, Abcam, Cambridge, CB, United Kingdom). Secondary antibodies coupled with Alexa Fluor 594-conjugated Goat Anti-Rabbit (1:200, AS039, Abclonal, Wuhan, China) were used, and nuclei counterstaining was performed using DAPI (1:1,000, Beyotime, Beijing, China) for 10 min at room temperature for cell nuclei staining. Subsequently, samples were washed with 0.5% PBST three times for 3 min each time and sealed with an anti-fluorescence quencher (P0126, Beyotime, Beijing, China).

### cAMP Levels Assay

To assess cAMP concentration, H9C2 cells were plated in 384-well plates at a density of 5,000/well and incubated overnight at 37°C; on the following day, the cell culture medium was replaced with drug-containing medium according to different groups. After completion of the addition, the cAMP level was quantified using the cAMP-Glo^™^ Assay Kit (#V1501, Promega, United States) following the manufacturer’s instructions.

### PKA Activity Assay

NRVMs (5 × 10^5^ cells/well) were isolated and seeded in a six well plate, following treatment with different agents, and total protein was harvested using lysis buffer containing phosphatase inhibitor and protease inhibitor. Immediately after, soluble proteins samples were obtained by taking the supernatant after centrifugation of the lysate at 12,000 rpm for 20 min at 4°C. Then, the supernatant was assayed for protein concentration by BCA (Beyotime, China) and their protein concentrations were all adjusted to 1 μl by lysis buffer. Finally, the supernatant protein samples’ PKA activity was measured by using ProFluor PKA assay (#V1240, Promega, United States) following the kit instructions.

### Enzyme-Linked Immunosorbent Assay

On completion of animal experiments, blood was collected in sterile tubes and then the mice were sacrificed immediately. After remaining still for 2 h in room temperature, the blood was centrifugated at 3,000 rpm for 10 min and the serum was separated. FUT8, β-1,4-mannosylglycoprotein 4-β-N-acetylglucosaminyltransferase (MGAT3), and MGAT4a levels in serum were measured by ELISA (ELISA kit for mouse FUT8, ELISA kit for mouse MGAT3, ELISA kit for mouse MGAT4a, MEIMIAN, China) according to the manufacturer’s instructions.

### Statistical Analysis

The data were shown as means ± standard deviations, and statistical analysis and plotting were conducted using GraphPad Prism 7 software (GraphPad, San Diego, CA, United States). All measurements were carried out at least three times. Two groups’ data analysis was compared using *t*-test, and multiple group comparisons were conducted using one-way ANOVA test. Statistically significant difference was set at *p* < 0.05.

## Results

### Sta Reduces Cardiac Remodeling Induced by ISO in Mice

**FIGURE 1:**
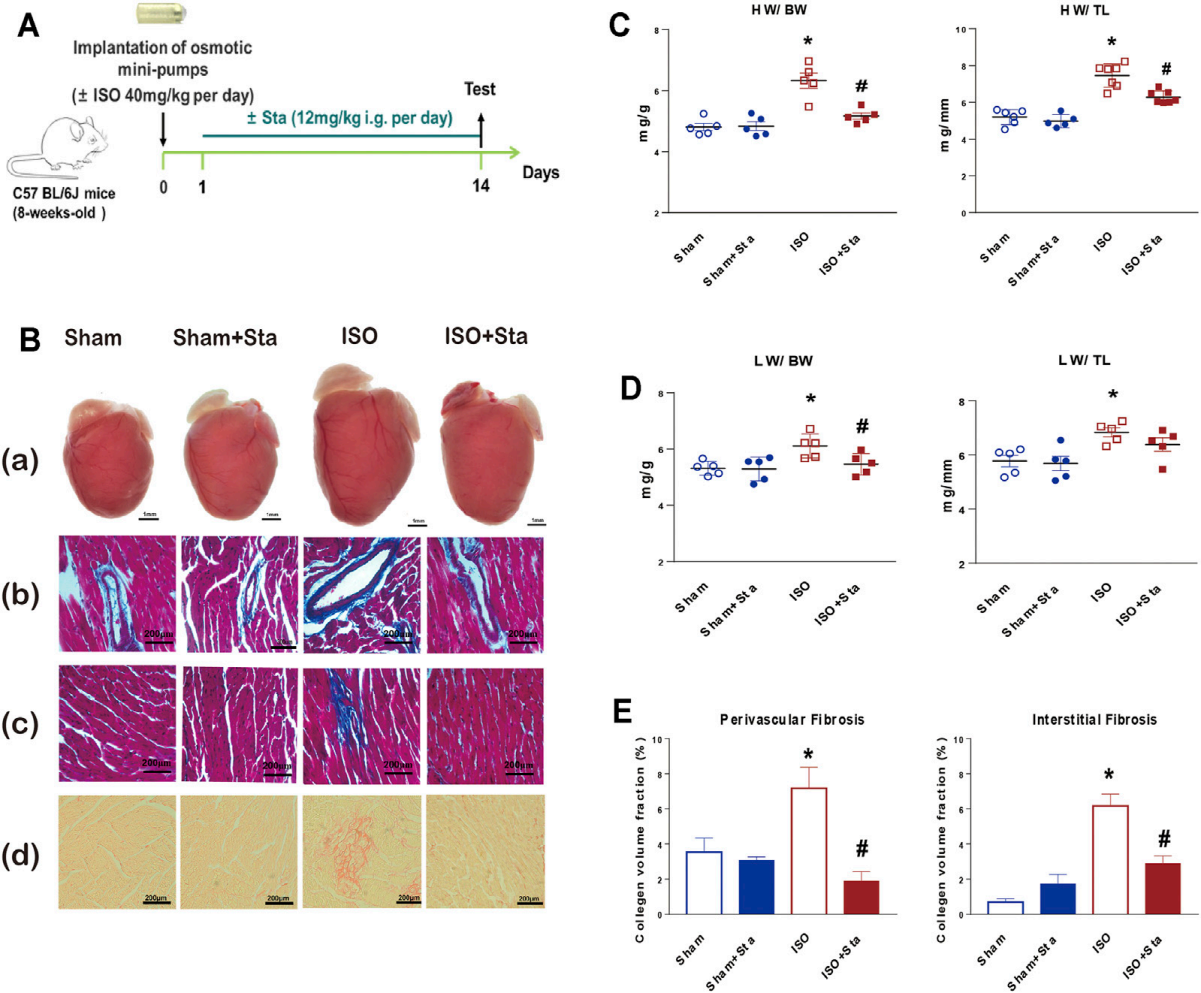
Stachydrine hydrochloride reduces cardiac remodeling induced by ISO in mice. **(A)** Timeline diagram of phenylephrine in mice. **(B)** Cardiac images: **(a)** comparison of heart size in mice that underwent Sham, Sham + Sta, ISO only, or ISO + Sta treatment; **(b, c)** collagen deposition was further recorded using Masson staining on 6-μm-thick heart sections; **(d)** collagen deposition was further recorded using Sirius red staining on 6-μm-thick heart sections. **(C)** HW:BW and HW:TL (*n* = 5 in each group). **(D)** LW:BW and LW:TL (*n* = 5 in each group). **(E)** Quantification of cardiac perivascular and interstitial fibrosis (*n* = 5 in each group). The data are expressed as mean ± standard error of mean, *p < 0.05 vs. Sham; ^#^ *p* < 0.05 vs. ISO. ISO, isoproterenol; Sta, stachydrine hydrochloride; HW, heart weight; BW, body weight; LW, lung weight; TL, tibial length.

C57BL/6J mice treated with either ISO or vehicle for 14 days in the presence of Sta (from day 1 to day 14) or its vehicle are shown in Figure 1A. Sta prevented ISO-induced increase in HW:BW, HW:TL, and LW/BW (Figures 1B(a)–D). Since fibrosis is an important factor in cardiac remodeling, Masson and picrosirius red staining were used to determine cardiac collagen deposition. Histological results showed that ISO significantly induced increased interstitial and perivascular fibrosis compared with the sham group. Sta treatment significantly suppressed cardiac fibrosis elicited by ISO (Figures 1B(b–d),E). HW/BW, HW/TL, LW/BW, LW/TL, and interstitial and perivascular fibrosis were similar at baseline in the vehicle and Sta-treated mice (Figure 1).

### Sta Modulates Hemodynamic Parameters During Chronic β1AR Activation *In Vivo*

**FIGURE 2:**
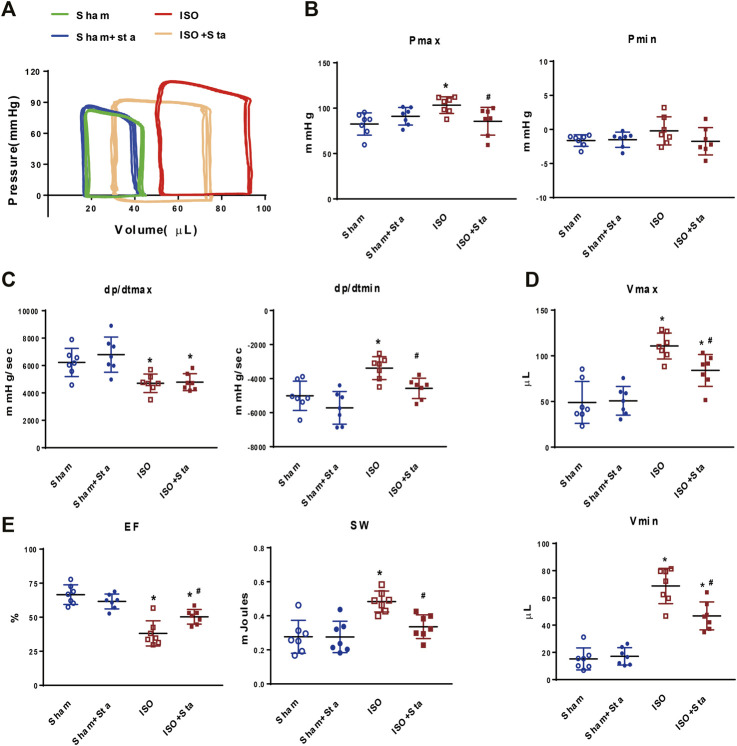
Stachydrine hydrochloride modulates hemodynamic parameters during chronic β1AR activation *in vivo*. **(A)** Representative images of hemodynamic parameters. **(B)** Quantification of Pmax and Pmin (*n* = 7 mice per experimental group). **(C)** Quantification of dP/dtmax and dP/dtmin (*n* = 7 mice per experimental group). **(D)** Quantification of Vmax and Vmin (*n* = 7 mice per experimental group). **(E)** Quantification of EF and SW (*n* = 7 mice per experimental group). Hemodynamic parameters were recorded and measured at 2 weeks after surgery. The data are expressed as mean ± standard error of mean, *p < 0.05 versus Sham; ^#^ *p* < 0.05 versus ISO. ISO, isoproterenol; Sta, stachydrine hydrochloride; Pmax, maximum left ventricular pressure; Pmin, minimum left ventricular pressure; dP/dtmax, peak rate of pressure rise; dP/dtmin, peak rate of pressure decline; Vmax, max of volume; Vmin, min of volume; EF: left ventricle ejection fraction; SW, stroke work.

According to PV loop analysis, ISO significantly elevated the left ventricular pressure (Pmax) and volume (Vmax and Vmin) and stroke work (SW). In addition, ISO induces a decline in cardiac systolic function (dP/dtmax and EF) and diastolic function (dP/dtmin). However, Sta treatment reduced the increase in the Pmax, Vmax, Vmin, dP/dtmin, and SW, and increased ISO-induced EF reduction (Figure 2). There was no significant difference in hemodynamic parameters between Sham and Sham + Sta groups.

### Transcriptome Analysis of Heart Tissue in Mice With ISO-Induced Heart Failure

**FIGURE 3:**
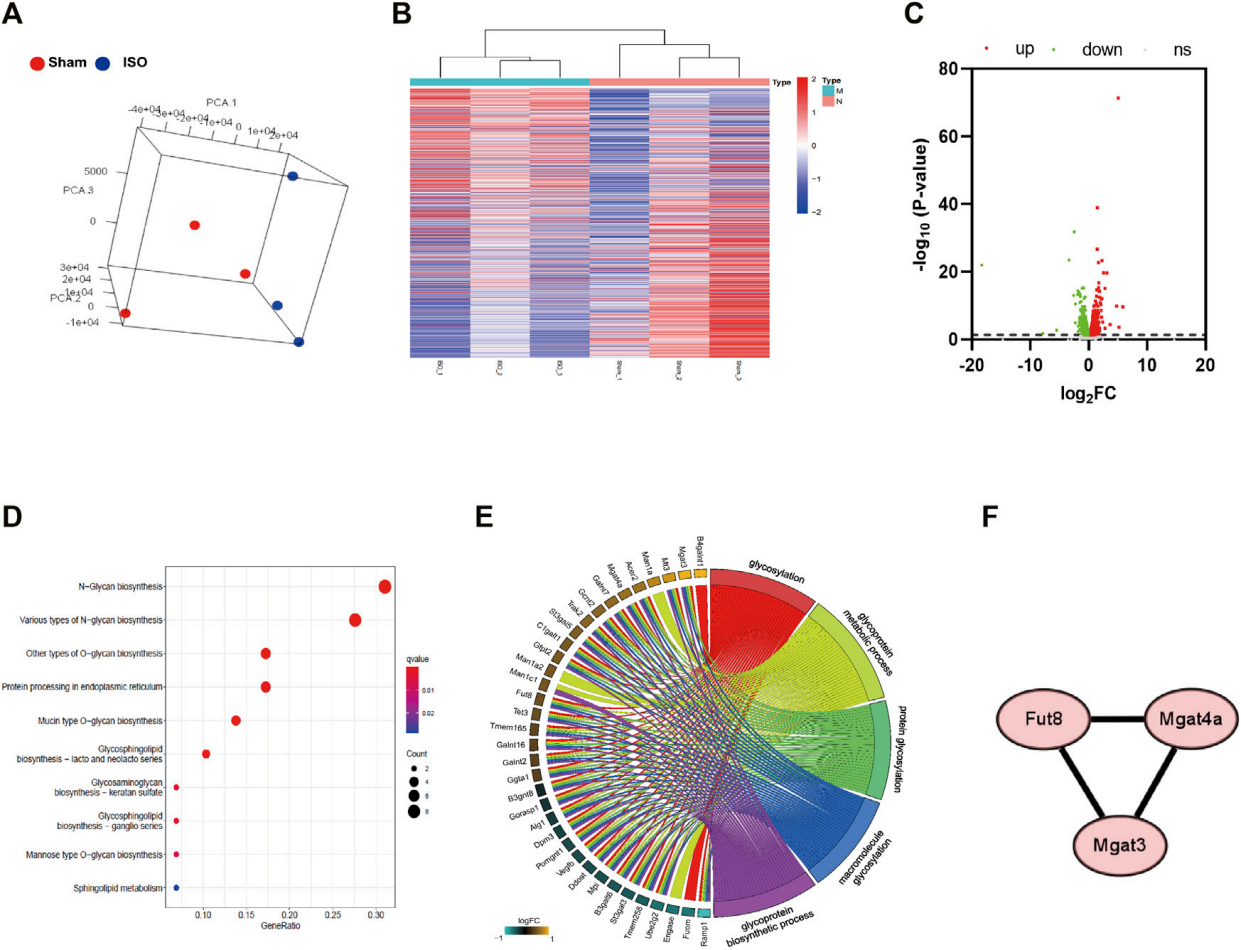
Transcriptome analysis of heart tissue in mice with ISO-induced heart failure. **(A)** Principal Componet Analysis to visualize similarities and differences among samples. **(B,C)** Heatmap and Volcano plot indicates upregulated and downregulated genes with differential mRNA abundance in the ISO group relative to the Sham group measured using RNA sequencing. **(D)** KEGG pathway analysis was performed by mapping N-type glycosylated genes in GO enrichment. **(E)** Gene regulation network was plotted based on the number of enriched genes in GO term. **(F)** Differential genes involved in transferase during N-glycosylation synthesis between Sham and ISO groups. *n* = 5 mice per experimental group. ISO, isoproterenol; Sta, stachydrine hydrochloride.

By conducting Principal Component Analysis (PCA) between the Sham and ISO groups, we found that the gene expression profile in each group could be clearly distinguished (Figure 3A). After comparing transcriptome analyses between Sham and ISO groups, 2,605 differentially expressed genes were identified, including 1,085 upregulated and 1,520 downregulated genes; then, the expression heatmap and volcano plot were presented in Figures 3B,C. Next, N-type glycosylated process category analysis was performed by mapping genes to GO enrichment; according to the results, 10 pathways were enriched significantly, especially in N-glycan-related processes (Figure 3D and Table 1). To assess the regulatory roles of the genes included in the GO process, gene regulation network was plotted based on the number of enriched genes in GO term (Figure 3E and Table 2). Then, on the foundation of STRING database and MCODE plugin, hub genes in the N-glycan GO process were scrutinized. Finally, FUT8, MGAT3, and MGAT4a were selected as the significantly differential genes in the N-type glycosylated process between Sham and ISO groups (Figure 3F).

**TABLE 1:** KEGG pathway analysis was performed by mapping N-type glycosylated genes in GO enrichment.

Id	Description	Gene ratio	Gene symbol
mmu00510	N-Glycan biosynthesis	9/43	Man1a/Fut8/Man1c1/Ddost/Man1a2/Mgat4a/Mgat3/Dpm3/Alg1
mmu00513	Various types of N-glycan biosynthesis	8/43	Man1a/Fut8/Man1c1/Ddost/St3gal3/Man1a2/Mgat4a/Alg1
mmu00514	Other types of O-glycan biosynthesis	5/43	St3gal3/C1galt1/Galnt16/Galnt2/Galnt7
mmu00512	Mucin type O-glycan biosynthesis	4/43	C1galt1/Galnt16/Galnt2/Galnt7
mmu00601	Glycosphingolipid biosynthesis—lacto and neolacto series	3/43	St3gal3/Gcnt2/Ggta1
mmu04141	Protein processing in endoplasmic reticulum	5/43	Man1a/Man1c1/Ddost/Ube2g2/Man1a2
mmu00533	Glycosaminoglycan biosynthesis—keratan sulfate	2/43	Fut8/St3gal3
mmu00604	Glycosphingolipid biosynthesis—ganglio series	2/43	St3gal5/B4galnt1
mmu00515	Mannose type O-glycan biosynthesis	2/43	St3gal3/Pomgnt1
mmu01250	Mannose type O-glycan biosynthesis	2/43	Gfpt2/Mpi
mmu00600	Sphingolipid metabolism	2/43	Acer2/B4galt6
mmu00520	Amino sugar and nucleotide sugar metabolism	2/43	Gfpt2/Mpi

**TABLE 2:** Gene regulation network was plotted based on the number of enriched genes in GO term.

Id	Description	Gene ratio	Gene symbol
GO:0070085	Glycosylation	31/43	Fut8/Ddost/Tmem258/Ube2g2/Gorasp1/Tmem165/Gfpt2/St3gal3/St3gal5/Mgat4a/Mgat3/Dpm3/Tet3/Vegfb/Acer2/C1galt1/Trak2/Galnt16/Mpi/Gcnt2/Alg1/B4galnt1/Pomgnt1/Ggta1/Galnt2/Galnt7/Mt3/Ramp1/B3galt6/B3gnt8/Fuom
GO:0009100	Glycoprotein metabolic process	33/43	Man1a/Fut8/Man1c1/Ddost/Tmem258/Ube2g2/Gorasp1/Tmem165/Gfpt2/St3gal3/Man1a2/St3gal5/Mgat4a/Mgat3/Dpm3/Tet3/Vegfb/Acer2/C1galt1/Trak2/Galnt16/Mpi/Gcnt2/Alg1/Pomgnt1/Ggta1/Galnt2/Galnt7/Mt3/Ramp1/B3galt6/B3gnt8
GO:0006486	Protein glycosylation	29/43	Fut8/Ddost/Tmem258/Ube2g2/Gorasp1/Tmem165/Gfpt2/St3gal3/St3gal5/Mgat4a/Mgat3/Dpm3/Tet3/Vegfb/Acer2/C1galt1/Trak2/Galnt16/Mpi/Gcnt2/Alg1/Pomgnt1/Ggta1/Galnt2/Galnt7/Mt3/Ramp1/B3galt6/B3gnt8
GO:0043413	Macromolecule glycosylation	29/43	Fut8/Ddost/Tmem258/Ube2g2/Gorasp1/Tmem165/Gfpt2/St3gal3/St3gal5/Mgat4a/Mgat3/Dpm3/Tet3/Vegfb/Acer2/C1galt1/Trak2/Galnt16/Mpi/Gcnt2/Alg1/Pomgnt1/Ggta1/Galnt2/Galnt7/Mt3/Ramp1/B3galt6/B3gnt8

### Sta Increases the N-Glycosylation of β1AR in the Heart of Mice With ISO-Induced Heart Failure

**FIGURE 4:**
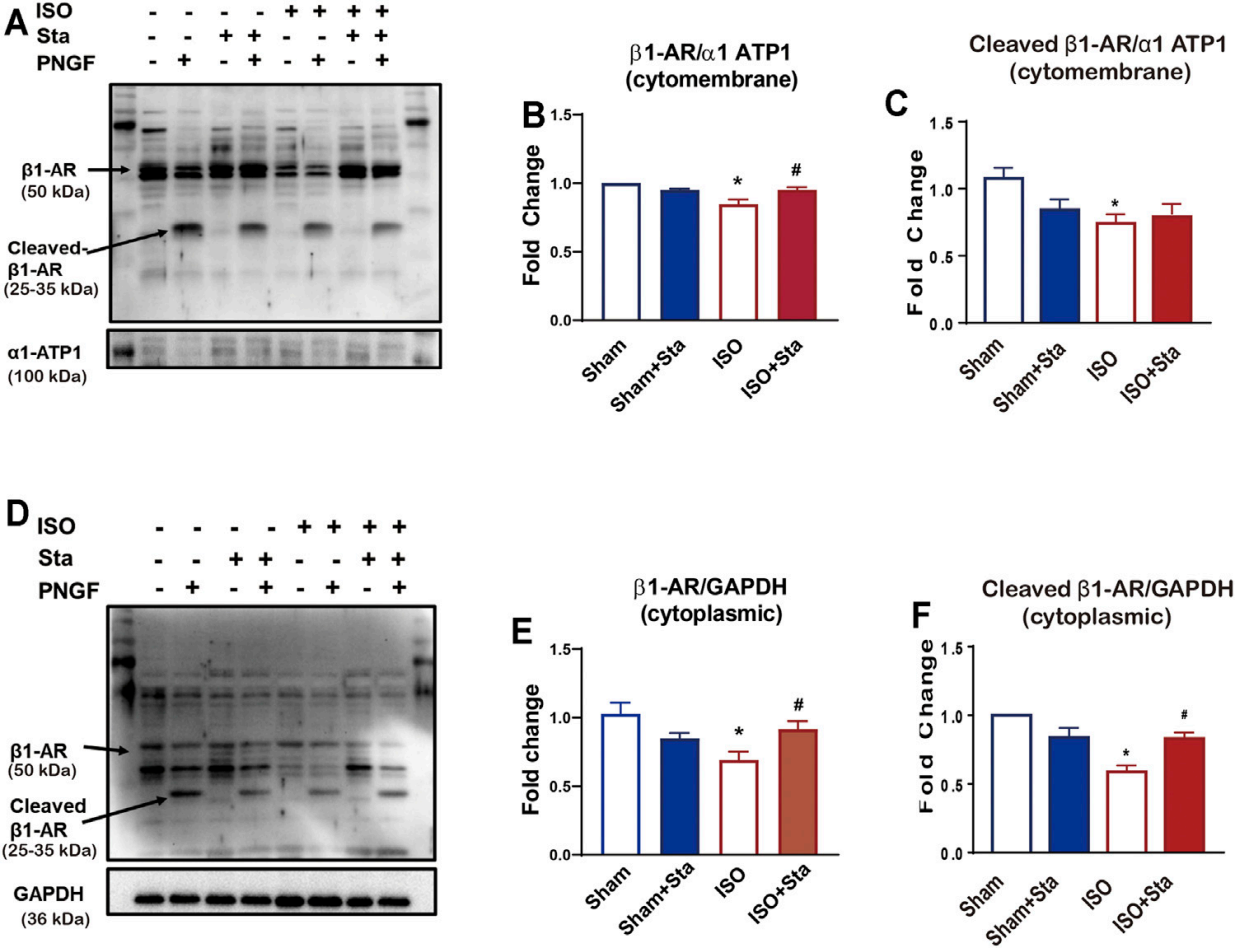
Stachydrine hydrochloride or Sta can increase the N-glycosylation of β1AR in the heart of mice with ISO-induced heart failure. **(A)** Representative Western blot of N-glycosylation of β1AR on the cell membrane of heart tissue after PNGase F digestion. **(B,C)** Quantification of β1AR and N-glycosylation of β1AR on the cell membrane. **(D)** Representative Western blot of N-glycosylation of β1AR in the cytoplasm of heart tissue after PNGase F digestion. **(E,F)** Quantification of β1AR and N-glycosylation of β1AR in cytoplasmic. *n* = 3 mice per experimental group. The data are expressed as mean ± standard error of mean, *p < 0.05 versus Sham; ^#^ *p* < 0.05 versus ISO. β1AR, β1 adrenergic receptors; ISO, isoproterenol; Sta, stachydrine hydrochloride.

β1AR plays an important role in the excitation–contraction coupling of the heart. In order to verify whether Sta can improve heart function by inhibiting the N-glycosylation of β1AR in ISO-induced heart failure mice, we performed Western blotting experiments in mice heart tissues. We separate the heart tissue protein into membrane protein and cytoplasmic protein to detect the N-terminal glycosylation expression of β1AR. There were multiple bands (antibody was polyclonal antibody), and the molecular weight of 50 kDa of β1AR was in accordance with the calculated molecular size. After PNGase F digestion, the approximately 25–35 kDa bands, in agreement with the monomer size, turned into one straight density band, indicating that the deglycosylated β1AR was mainly retained as a monomer (Figure 4). As shown in Figures 4B,C, the expression of β1AR and N-terminal glycosylation of β1AR on the cell membrane of the ISO group were decreased (*p* < 0.05). Among the cytoplasmic proteins, the expression of β1AR (Figures 4E) and N-glycosylation of β1AR (Figure 4F) was also downregulated in the ISO group (*p* < 0.05), while stachydrine hydrochloride can increase the N-glycosylation of β1AR induced by ISO (*p* < 0.05). Although Sta cannot significantly increase the expression of N-glycosylation of β1AR on the cell membrane surface, it can indeed maintain the number of β1AR on the cytoplasm and cell membrane (*p* < 0.05).

### The N-Glycosylation of β1AR Decreased After Continuous ISO Stimulation, While Sta Increases Its Expression *In Vitro*

**FIGURE 5:**
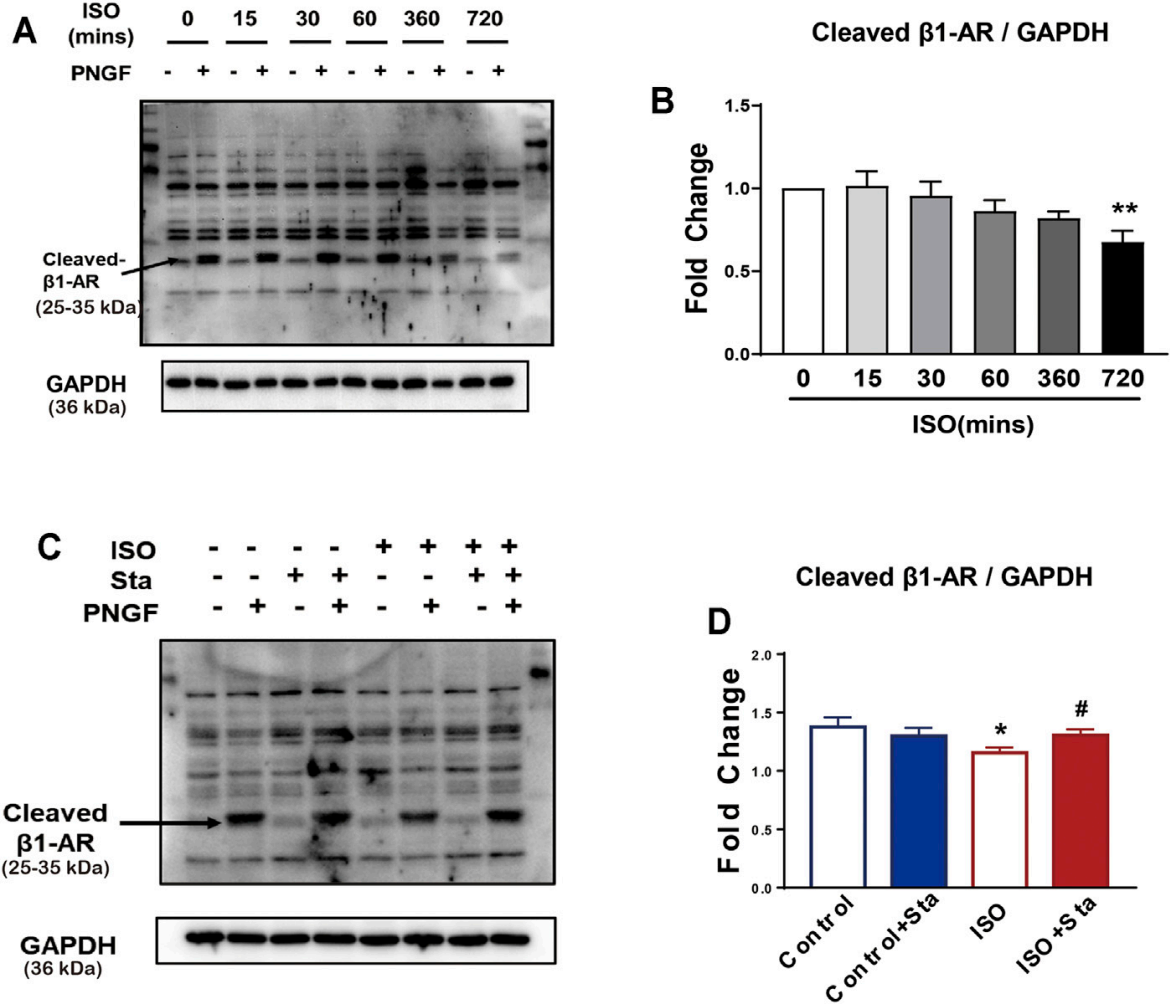
The N-glycosylation of β1AR decreased after continuous ISO stimulation, while stachydrine hydrochloride can increase its expression *in vitro*. **(A)** Representative Western blot of N-glycosylation of β1AR after PNGase F digestion exposed to ISO at different time points in NRVMs (from three independent experiments). **(B)** Quantification of cardiac N-glycosylation of β1AR. **(C)** Representative Western blot of Sta on N-glycosylation of β1AR exposed to ISO at 720 min (from three independent experiments). **(D)** Quantification of cardiac N-glycosylation of β1AR. The data are expressed as mean ± standard error of mean, *p < 0.05 versus control; ^#^ *p* < 0.05 versus ISO. β1AR, β1 adrenergic receptors; ISO, isoproterenol; Sta, stachydrine hydrochloride; NRVMs, neonatal rat ventricular myocytes.

To explore the time–effect relationship of ISO (0.1 μMol) on N-glycosylation, we detected the expression of N-glycosylation at different time points (0, 15, 30, 60, 360, and 720 min) of ISO in NRVMs. Western blotting for β1AR in whole cell lysate at different time points of ISO treatment. The results showed that N-glycosylation was time-dependent, and the longer the time, the more N glycosylation. Compared with 0 min, the N-glycosylation of β1AR of ISO in 720 min was significantly decreased (*p* < 0.05) (Figures 5A,B). The diminution of N-glycosylation of β1AR induced by ISO stimulation for 720 min was inhibited by Sta (10 μMol) (*p* < 0.05) (Figures 5C,D).

### Decreased N-Glycosylation of β1AR Will Downregulate the β1AR/cAMP/PKA Signal Pathway and Inhibit Myocardial Excitation and Contraction Coupling

**FIGURE 6:**
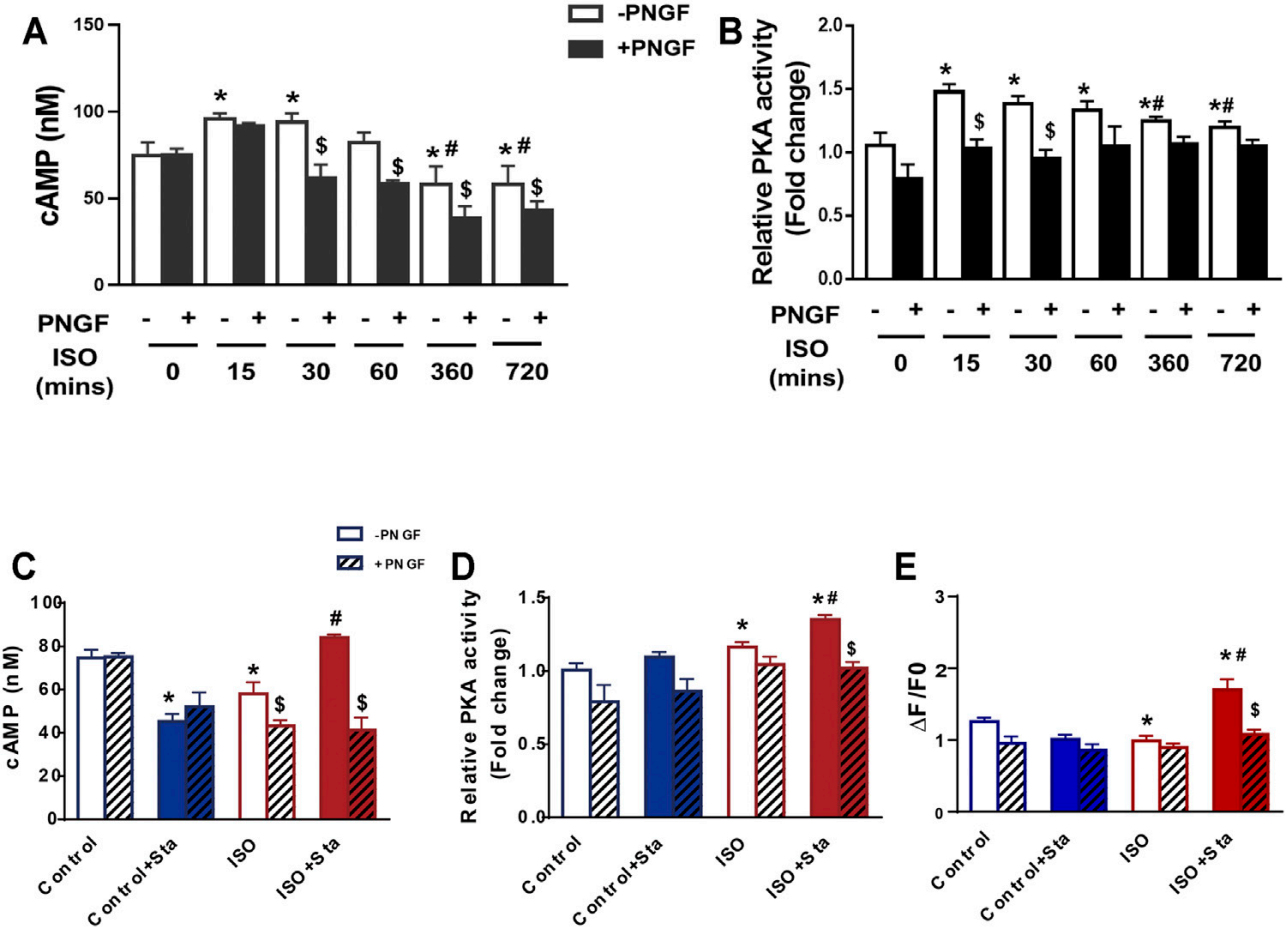
Decreased N glycosylation of β1AR will downregulate the β1AR/cAMP/PKA signal pathway. **(A)** Quantification of cAMP production after PNGase F digestion exposed to ISO at different time points in H9C2 cells (from three independent experiments). **(B)** Quantification of PKA activity after PNGase F digestion exposed to ISO at different time points in NRVMs (from three independent experiments). **(C,D)** Quantification of Sta on cAMP production and PKA activity exposed to ISO at 720 min (from three independent experiments). **(E)** Quantification of Ca^2+^ transient parameters exposed to ISO at 720 min in AMVMs (*n* = 15 cells from 3 mice in each group). The data are expressed as mean ± standard error of mean, *p < 0.05 versus control; ^#^ *p* < 0.05 versus ISO; $*p* < 0.05 versus -PNGF. AMVMs, adult mouse ventricular myocytes; ISO, isoproterenol; Sta, stachydrine hydrochloride; NRVMs, neonatal rat ventricular myocytes.

**FIGURE 7:**
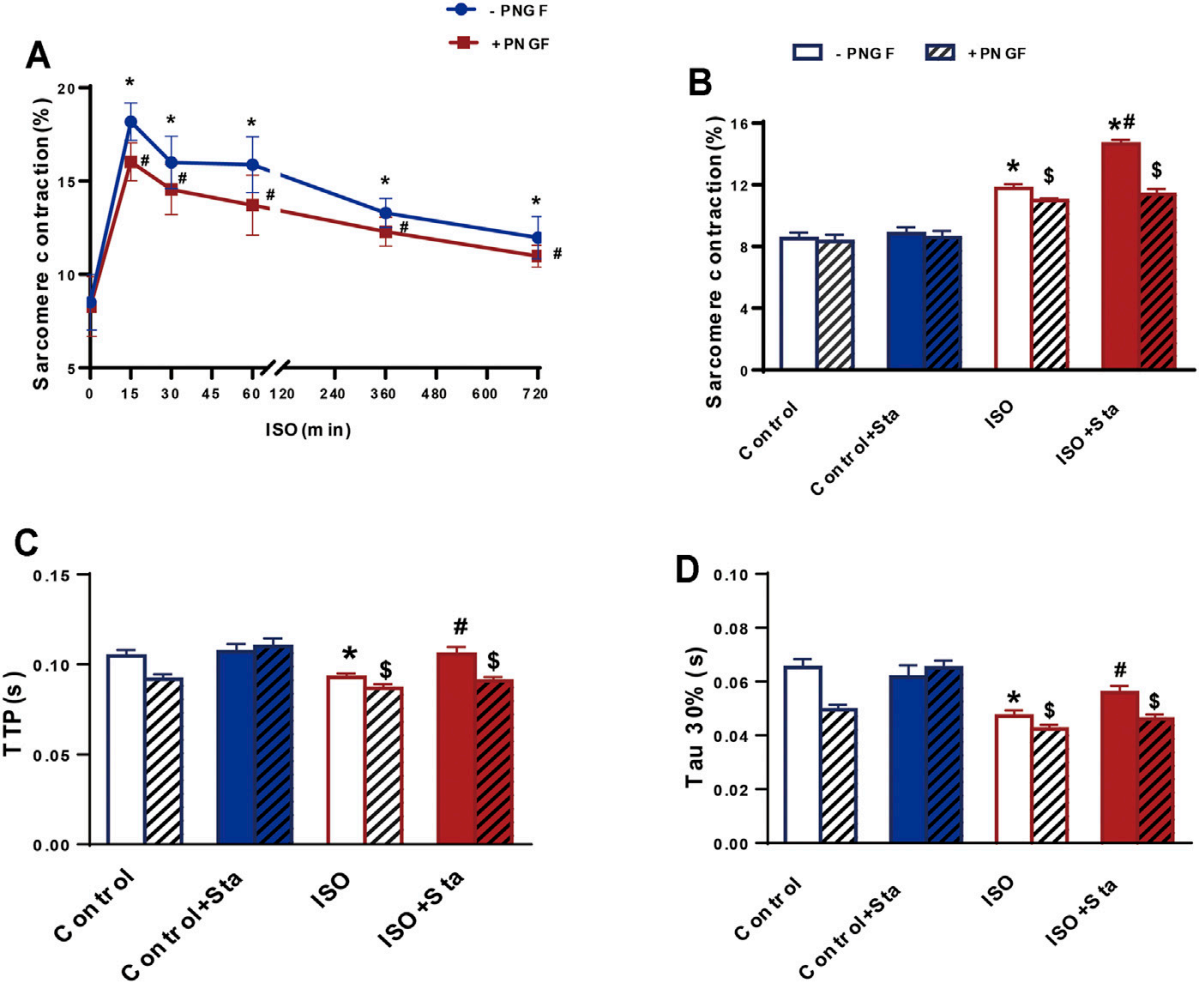
Stachydrine hydrochloride improves myocardial contraction and diastolic function decline caused by continuous ISO stimulation by regulating N-glycosylation. **(A)** Quantification of sarcomere contraction after PNGase F digestion exposed to ISO at different time points in AMVMs (*n* > 30 cells from 3 mice in each group). **(B–D)** Quantification of Sta on sarcomere contraction, TTP and Tau 30% in AMVMs exposed to ISO at 720 min (*n* > 20 cells from 3 mice in each group). The data are expressed as mean ± standard error of mean, *p < 0.05 versus control; #*p* < 0.05 versus ISO; $*p* < 0.05 versus -PNGF. AMVMs, adult mouse ventricular myocytes; ISO, isoproterenol; Sta, stachydrine hydrochloride; Tau, relaxation time constant; TTP, time to peak.

We conducted the following experiments: we detected the cAMP expression and PKA activity in NRVMs and myocardial systolic function in AMVMs at different time points (0, 15, 30, 60, 360, and 720 min). The results showed that with the prolongation of ISO stimulation time, the content of cAMP, activation of PKA, and amplitude of sarcomere contraction were decreased (*p* < 0.05). After PNGase F is added, the content of cAMP, activation of PKA, and amplitude of sarcomere contraction decrease rapidly (*p* < 0.05) (Figures 6A,B and Figure 7A).

We tested the effects of Sta on cAMP, PKA, calcium transients, and myocardial contraction and diastolic function when ISO was applied for 720 min. Compared with the ISO group, the ISO + Sta group can significantly increase the cAMP content, PKA activity, and amplitude of calcium transient (*p* < 0.05). After adding PNGase F, the effect of Sta on the increase of cAMP, PKA, and Ca^2+^ transients disappeared (*p* < 0.05) (Figures 6C–E). The downstream biological effects of cAMP/PKA/Ca^2+^ signaling pathway regulation are myocardial contraction and diastolic function. Figures 7B–D show that Sta can slow down the decrease in sarcomere contraction amplitude, time to peak (TTP), and sarcomere relaxation time constant (Tau) caused by continuous ISO stimulation (*p* < 0.05). After adding PNGase F to shear N-glycosylation, these effects of Sta disappear (*p* < 0.05).

### Sta Significantly Reduced ISO-Induced Cardiac N-Linked Glycoproteins With α-1,6-Fucosylation

**FIGURE 8:**
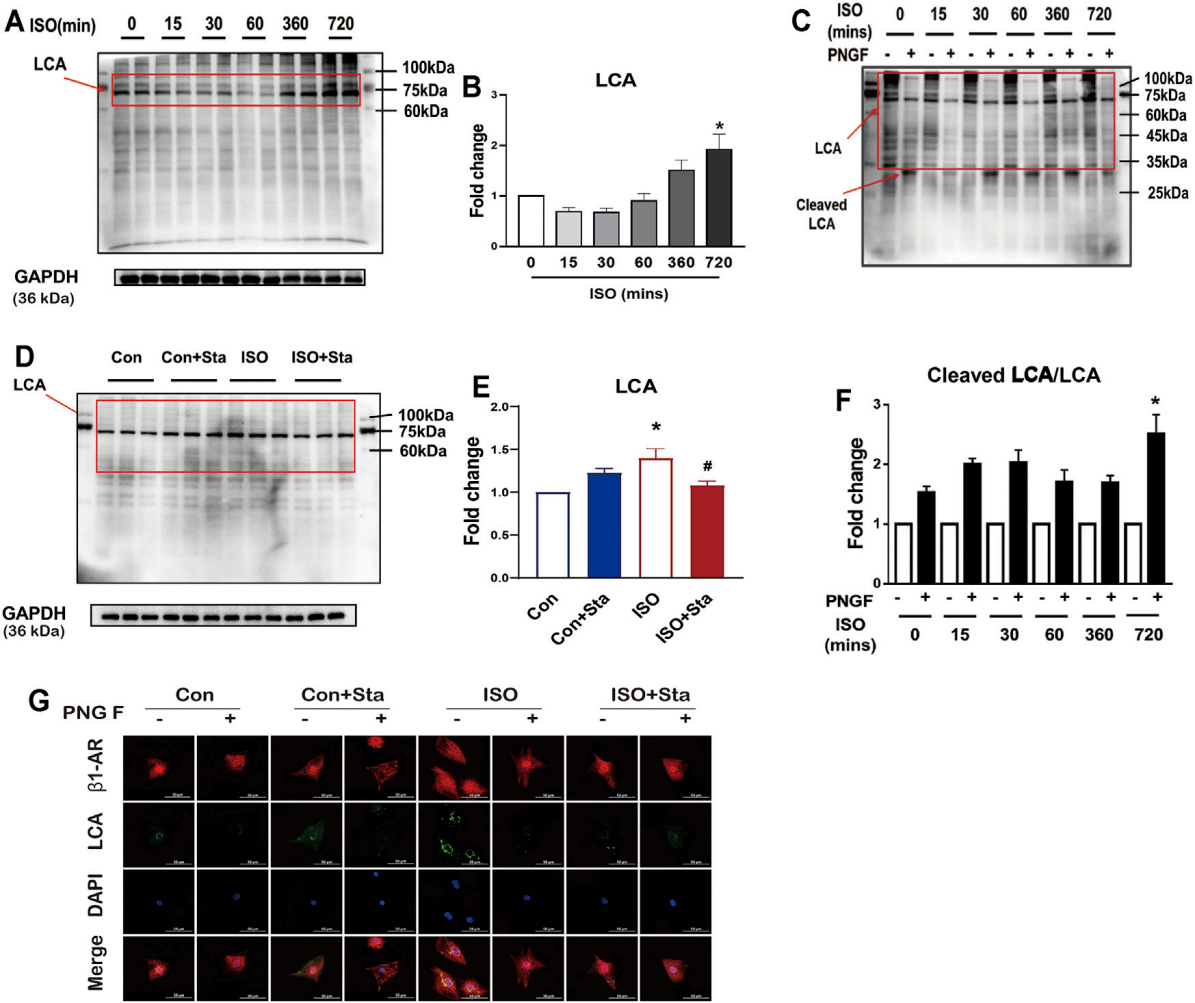
Stachydrine hydrochloride reduced ISO-induced cardiac N-linked glycoproteins with α-1,6-fucosylation *in vitro*. **(A,B)** Representative Western blot and quantification of LCA exposed to ISO at different time points in NRVMs (from three independent experiments). **(C,D)** Representative Western blot and quantification of LCA after PNGase F digestion exposed to ISO at different time points in NRVMs (from three independent experiments). **(E,F)** Representative Western blot and quantification of Sta on cAMP production and PKA activity exposed to ISO at 720 min (from three independent experiments). **(G)** Representative immunofluorescence of LCA and β1AR exposed to ISO at different time points in NRVMs. The data are expressed as mean ± standard error of mean, *p < 0.05 versus control; ^#^ *p* < 0.05 versus ISO. ISO, isoproterenol; Sta, stachydrine hydrochloride; NRVMs, neonatal rat ventricular myocytes.

**FIGURE 9:**
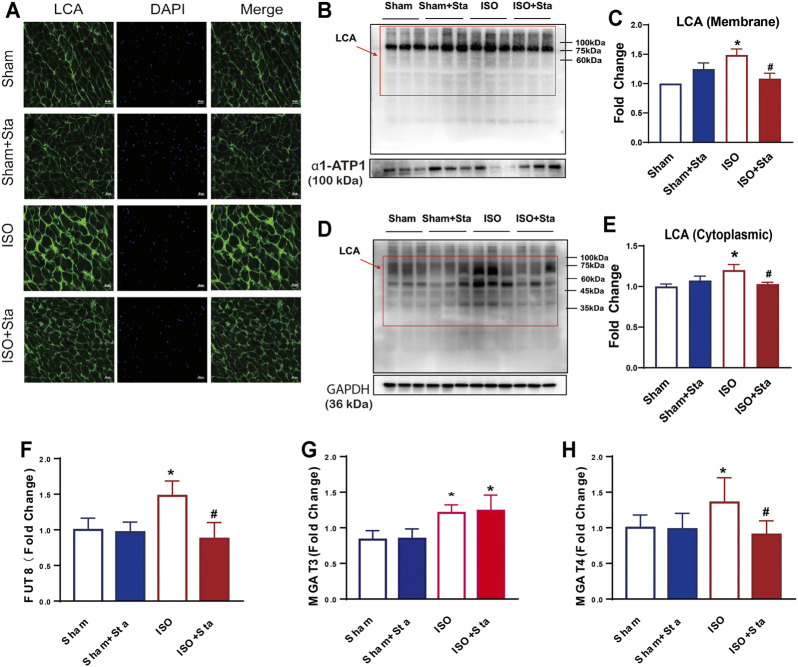
Stachydrine hydrochloride inhibits the expression of FUT8 and MAGT4a to reduced α-1,6-fucosylation. **(A)** Images of FITC-labeled Lens culinaris agglutinin (LCA) lectin affinity fluorescence histopathology sections. **(B–E)** Representative Western blot and quantification of LCA exposed to ISO at 2 weeks on the cell membrane and in cytoplasmic. **(F–H)** FUT8, MGAT3, and MGAT4a expression measurement with ELISA kits. *n* = 5 mice per experimental group. The data are expressed as mean ± standard error of mean, *p < 0.05 versus Sham; ^#^ *p* < 0.05 versus ISO. FUT8, α-1,6-fucosyltransferase; LCA, Lens culinaris agglutinin; MGAT3, β-1,4-mannosylglycoprotein 4-β-N-acetylglucosaminyltransferase; MGAT4a.α-1,3-mannosyl-glycoprotein 4-β-N-acetylglucosaminyltransferase A; ISO, isoproterenol; Sta, stachydrine hydrochloride.

LCA is an important tool for the study of glycoproteins with N-linked glycans; the main glycoproteins that are profiled by lectins are core α-1,6-fucose glycans. To verify the expression differences in N-linked glycoproteins with α-1,6-fucosylation, lectin affinity histochemical analysis is presented in Figures 8, 9.

As shown in Figures 8A,B, with the prolongation of ISO action time, the α-1,6-fucosylation levels gradually increased. Compared with 0 min, the α-1,6-fucosylation expression level of ISO in 720 min was significantly increased (*p* < 0.05). To measure the degree of N-linked glycoproteins with α-1,6-fucosylation, we measured the ratio of cleaved LCA to LCA. Then, the control group without PNGase F was homogenized. We also found that after 720 min of ISO action, N-linked glycoproteins with α-1,6-fucosylation increased significantly (*p* < 0.05) (Figures 8C,D). The increase of α-1,6-fucosylation levels induced by ISO at 720 min was significantly reduced by stachydrine hydrochloride (*p* < 0.05) (Figures 8C,D). The lectin affinity histochemical analysis on NRVMs is shown in Figure 8G. α-1,6-fucosylation is mainly distributed around the nuclear membrane. Compared with the control group, the fluorescence brightness of α-1,6-fucosylation increased after 720 min of ISO exposure, indicating that the expression of α-1,6-fucosylation increased. Sta can inhibit the increase in α-1,6-fucosylation induced by ISO. However, after adding PNGase F, the green fluorescence around the nuclear membrane of each group disappeared.

Images of FITC-labeled LCA lectin affinity fluorescence histopathology sections. All the images of the sections showed that the extracellular and interstitial matrix were obviously stained. In addition, we found that the green fluorescence of LCA staining was coincident with the blue fluorescence of the nucleus. From the fluorescence intensity, we can see that the α-1,6-fucosylation in the ISO group myocardium was upregulated significantly compared with the sham group. Compared with the ISO group, the fluorescence of the ISO + Sta group was relatively weak (Figure 9A). In order to determine the distribution of α-1,6-fucosylation in cells, we extracted cytoplasmic proteins and cell membrane proteins. Through LCA lectin blot, we found that the expression of α-1,6-fucosylation (both cytoplasmic proteins and membrane proteins) in the ISO group increased, while Sta could inhibit the increase of myocardium protein α-1,6-fucosylation residues on N-glycosylation (*p* < 0.05) (Figures 9B–E). Consistent with the results of transcriptome analysis, the expression of FUT8, MAGT3, and MAGT4a in the serum of ISO mice was upregulated, while Sta could inhibit the expression of FUT8 and MAGT4a (*p* < 0.05) (Figures 9F,G).

## Discussion

Protein glycosylation is a posttranslational modification essential for protein functions such as proper folding, targeting to cellular compartments, and modulating receptor/ion channel activities (Ohtsubo and Marth, 2006; Ednie et al., 2019). The oligosaccharides of cell surface glycoproteins can regulate recognition processes, such as signal transduction, cell adhesion, immune response, and host–pathogen interactions. If the glycoprotein changes slightly, it will change the biological function (Zhao et al., 2018). In this study, we first found that Sta can inhibit ISO-induced cardiac hypertrophy and improve cardiac function in mice with ISO-induced heart failure. Then, through transcriptomics analysis, we found that many N-glycosylation regulatory signaling pathways in the ISO group have changed. Therefore, our hypothesis is whether Sta improves heart function by regulating N-glycosylation.

There are three subtypes of βAR: β1AR, β2AR, and β3AR. All of these subtypes couple to Gs and therefore increase cellular cAMP levels when stimulated with agonist (Steinberg, 2018; Alhayek and Preuss, 2021). β1AR is found in a variety of tissues but is particularly highly expressed in the heart, where it mediates the bulk of the effects of epinephrine on cardiac function. Glycosylation modulates interactions of receptors and ligands with themselves, coregulatory molecules, and distinct membrane domains of intact cells, thereby altering signal transduction (Ednie et al., 2019). Studies have reported that blocking the N-glycosylation of β1AR will reduce the potency of isoproterenol in cyclic AMP generation assays, and reduce the expression of β1AR on the cell surface and dimerization (He et al., 2002). In the ISO model, we found that the expression of β1AR in the myocardial cell membrane and cytoplasm decreased. At the same time, we also found that the β1AR N-glycosylation of cell membrane proteins and cytoplasmic proteins of heart tissues in the ISO group was decreased significantly. In addition, we verified that continuous ISO stimulation can reduce the N-glycosylation level of β1AR *in vitro*, which is consistent with the results of previous reports.

*In vivo* experiments revealed that Sta can increase the reduction of β1AR in the cell membrane and cytoplasm induced by ISO, and Sta can increase the N-glycosylation level of β1AR in the cytoplasm. *In vitro* experiments also found that Sta can increase the decrease in N-glycosylation of β1AR induced by ISO. Does Sta improve the cardiac function of the ISO model related to maintaining the amount of β1AR and regulating N-glycosylation?

We know that the β1AR/cAMP/PKA signaling pathway plays an important role in the coupling of myocardial excitation and contraction. Is the decline in heart function caused by long-term continuous stimulation of ISO related to the decreased expression of N-glycosylation on β1AR? Subsequently, we tested whether the downregulation of N-glycosylation on β1AR affects its downstream cAMP/PKA signaling pathway. Continuous stimulation of ISO caused the downregulation of cAMP, PKA, and myocardial contractile function, and the downregulation trend was consistent with the downregulation trend of N-glycosylation on β1AR.

After cutting or not cutting N-glycosylation with PNGase F, we observed the effect of ISO on cAMP, PKA, and myocardial contractile function at different times. We found that when the N-glycosylation is not sheared, the effect of prolonging the action time of ISO on the cAMP, PKA, and myocardial contractile function is to promote first, and then gradually decreases, with the lowest at 720 min. The above results indicate that the N-glycosylated cleavage of the β1AR receptor will affect the contraction and relaxation of the downstream cardiomyocytes. After shearing N-glycosylation, the shrinkage of ISO decreases even more. Sta can increase the cAMP, PKA, calcium signal, and the downregulation of myocardial contraction and relaxation function caused by the continuous stimulation of ISO. However, after cutting N-glycosylation, the protective effect of Sta also disappeared. Therefore, we infer that the protective effect of Sta on the β1AR/cAMP/PKA signaling pathway is achieved by upregulating the N-glycosylation of β1AR. What is the mechanism by which Sta regulates N-glycosylation?

LCA is an important tool for the study of glycoproteins with N-linked glycans (Zhao et al., 2018). This lectin recognizes sequences containing α-1,6-fucose structures and also identifies additional sugars as part of the receptor structure (André et al., 2009). Studies have shown that the upregulation of α-1,6-fucosylation expression can cause abnormalities in cardiac structure and function (Zhao et al., 2018). To determine whether Sta improves cardiac function by regulating α-1,6-fucosylation, we use LCA lectin to analyze the differences in glycans between groups. LCA lectin electrophoresis showed that the α-1,6-fucosylation expression of cardiomyocytes and tissues increased significantly after continuous ISO stimulation, and the N-glycosylation of both cell membrane proteins and cytoplasmic proteins was significantly increased. We speculate that the upregulation of α-1,6-fucosylation may result in the downregulation of N-glycosylation on β1AR. Sta can inhibit the increase in α-1,6-fucosylation induced by ISO, thereby increasing the number of β1AR, maintaining the basis of cAMP/PKA signaling pathway, and improving myocardial function.

There are several N-glycan branching enzymes such as N-acetyl-glucosaminyltransferases GnT-I, II, III, IV, V, VI, IX (Vb), and FUT8, and a modifying enzyme, β-galactoside α-2,6 sialyltransferase 1. These enzymes are intimately associated with various diseases and biological functions by modifying target proteins (Taniguchi et al., 2021). Through transcriptome analysis, we found that among the enzymes related to N-glycosylation in the ISO model, the gene expression levels of FUT8, MAGT3, and MAGT4a increased significantly. Does Sta inhibit α-1,6-fucosylation by inhibiting these three transferases? We found that Sta could inhibit the expression of FUT8 and MAGT4a.

This study verified that when ISO was continuously stimulated, the expression of N-glycosylation on β1AR gradually decreased, the expression of LCA linked to N-glycosyl gradually increased, and cAMP/PKA and downstream signaling pathways gradually decreased. We have also verified the effect of downregulation of N-glycosylation on β1AR on cAMP/PKA and downstream signaling pathways. However, how the upregulation of LCA directly causes downregulation of N-glycosylation on β1AR has not been verified. This is also a technical difficulty. This is the shortcoming of this research.

## Conclusion

In summary, we determined the contribution of Sta in isoproterenol-induced heart failure through α-1,6-fucosylation linked by N-glycosylation. The underlying mechanism may be related to the inhibition of the synthesis of α-linked mannose residues on the N-terminal sugar chain by reducing FUT8 and MGAT4a. Our results reveal the molecular mechanism of Sta in the prevention and treatment of heart failure, and highlight the important role of N-glycosylation on β1AR in the progression of heart failure. It provides a new idea for elucidating the cardiovascular protective effect of the traditional Chinese medicine monomer Sta.

## Data Availability

The transcriptome library construction and sequencing data presented in the study was deposited in the NCBI SRA BioProject repository, and the accession number was PRJNA793393.

## Abbreviations

β1AR, β1 adrenergic receptors; AMVMs, adult mouse ventricular myocytes; BW, body weight; cAMP, cyclic adenosine monophosphate; dP/dtmax, peak rate of pressure rise; dP/dtmin, peak rate of pressure decline; EF, ejection fraction; ELISA, enzyme-linked immunosorbent assay; FUT8, α-1,6-fucosyltransferase; GPCRs, G protein-coupled receptors; HW, heart weight; ISO, isoproterenol; LCA, lens Culinaris Agglutinin; LW, lung weight; MGAT3, β-1,4-mannosylglycoprotein 4-β-N-acetylglucosaminyltransferase; MGAT4a.α-1,3-mannosyl-glycoprotein 4-β-N-acetylglucosaminyltransferase A; NRVMs, neonatal rat ventricular myocytes; PBS, phosphate-buffered saline; PCA, principal component analysis; Pmax, maximum left ventricular pressure; Pmin, minimum left ventricular pressure; PNGF, PNGase F; PKA, protein kinase A; Sta, stachytine hydrochloride; SW, stroke work; Tau, relaxation time constant; TL, tibial length; TTP, time to peak; Vmax, max of volume; Vmin, min of volume.
